# A diverse semisynthetic humanized scFv phage display library for anti-CXCL16 antibodies

**DOI:** 10.1016/j.jbc.2025.110692

**Published:** 2025-09-08

**Authors:** ZhenSheng Li, Qi Chen, Shihui Wang, JianFeng Chen, ShiYang Chen

**Affiliations:** 1 Key Laboratory of Systems Health Science of Zhejiang Province, School of Life Science, Hangzhou Institute for Advanced Study, University of Chinese Academy of Sciences, Hangzhou, China; 2 Key Laboratory of Multi-Cell Systems, Shanghai Institute of Biochemistry and Cell Biology, Center for Excellence in Molecular Cell Science, Chinese Academy of Sciences, Shanghai, China

**Keywords:** antibody library, phage display, antibody screening, Kunkel mutagenesis, CXCL16

## Abstract

Phage display libraries of human single-chain variable fragments (scFvs) serve as a valuable resource for generating fully human antibodies for scientific and clinical applications. In this study, we designed and constructed a highly diverse semisynthetic humanized scFv phage display library using an optimized Kunkel mutagenesis approach. Our optimizations eliminated residual template, enhancing mutagenesis efficiency and expanding library diversity with a reservoir capacity exceeding 10^10^. For this semisynthetic library, the complementarity-determining region 3 was structurally designed to mimic the natural human antibody repertoires, encompassing comprehensive sequence variability and length distributions. To functionally characterize this antibody library, we performed targeted screening against CXC motif chemokine ligand 16 (CXCL16), a crucial chemokine involved in inflammatory pathogenesis and tumor microenvironment regulation. We successfully identified multiple CXCL16 specific antibodies, one of which demonstrated strong blocking activity for the CXCL16-CXCR6 axis. Structural modeling and docking analyses further elucidated the key binding sites of CXCL16 and the antibody, offering insights for future antibody optimization. These results highlight the potential of this humanized scFv library as a robust platform for therapeutic antibody discovery, with broad applications in diagnostics and biomedical research.

Over the past decades, phage display technology (PDT) has been widely used for generating peptide affinity-specific humanized antibodies, which provides a revolutionary new approach for antibody discovery and enables the preparation of fully humanized monoclonal antibodies (mAbs) (1). The major advantage of fully humanized mAbs is their efficient manipulation of effector functions while circumventing the immunogenicity associated with rodent antibodies (2). The production of humanized mAbs for clinical and research purposes is directly linked to advances in PDT (3, 4, 5, 6). To date, PDT has become a major source of humanized antibodies and successfully produced nearly 20 marketed therapeutic mAbs, such as adalimumab, atezolizumab, and ranibizumab, as well as numerous others undergoing preclinical or clinical research (7).

Phage display libraries are classified into three types based on the source of their antibody gene sequences: immune libraries, naive libraries, and synthetic libraries (8, 9). Immune libraries generate high-affinity binders for specific targets through the immunization of animals with specific antigens (10). However, this method is time-consuming and uncertain, as it necessitates preparing distinct antibody libraries for different antigens (11). Furthermore, it may not generate antibodies against weak antigens or autoantigens (9, 12). Naïve antibody libraries are constructed using B cells from unimmunized or healthy donors (13). Although these libraries can be quickly screened for a variety of unknown antigens, the quality of naïve antibody libraries is significantly influenced by the genetic background of the donor, resulting in inherent limitations regarding controllability, capacity, and diversity (9, 11, 14). The synthetic libraries are designed with high stability scaffolds and one or more mutated complementarity-determining regions (CDRs) using oligonucleotide-directed mutagenesis. According to the proportion of artificially synthesized variable regions in the antibody sequences, synthetic antibody libraries are categorized into semisynthetic and fully synthetic libraries. Engineered semisynthetic and synthetic libraries usually need to have large capacity and high diversity to screen high-affinity binders and can rapidly generate antibodies against broad antigens without conducting animal experiments. The mutation process and the sequences can be precisely controlled and optimized to produce high expression levels of antibodies (9, 15).

Besides intact full length antibodies, the synthetic libraries have been further extended to the display and selection of small antibody fragments, such as single-chain variable fragment (scFv), fragment antigen-binding (Fab), and variable heavy chain domain (VHH), leading to broad applications for antibody discovery and development across medicine and scientific research (16, 17, 18). ScFv platform is routinely used for phage display due to its efficient production in bacterial systems and typically exhibits greater stability and solubility than other antibody formats (19). ScFv is an engineered antibody fragment that consists of the variable domains of the heavy (VH) and light (VL) chains, connected by a short peptide linker (typically 15–20 amino acids) (20, 21). Structurally, the VH and VL domains fold together to form a functional antigen-binding site, maintained by noncovalent interactions. Each domain contains three CDRs, which are primarily responsible for antigen recognition. The flexible linker ensures proper orientation of the variable domains while preventing misfolding or aggregation. ScFv features multiple antigen-binding sites with smaller molecular size (25–27 kDa), amplifying their proficiency in recognizing complex epitopes and can be easily reformatted into other antibody fragments, such as minibody or scFv-Fc (19, 22, 23). Several strategies have been employed to design and construct synthetic libraries with high functional diversity based on the mutagenesis method originally developed by Kunkel *et al.* (1985) (24, 25, 26, 27). This approach mutates one or more CDRs by introducing oligonucleotides to obtain a large number of mutant plasmids, which could rapidly and efficiently produce a large capacity of synthetic antibody libraries. However, low experimental efficiency and original template residue still exist for this method (26, 27, 28, 29, 30, 31). Here, we optimized the Kunkel method and designed a humanized scFv phage display library with extensive amino acid (AA) and length diversity of CDR3. The amino acid diversity in this library closely mimics the distribution found in natural human antibodies, and it has a reservoir capacity exceeding 10^10^. To evaluate the library’s potential for specific antibody screening, we performed panning against CXC motif chemokine ligand 16 (CXCL16), a pivotal α-chemokine subfamily member implicated in tumorigenesis, atherosclerosis, renal fibrosis, and nonalcoholic fatty liver disease (32, 33, 34, 35). Ultimately, 12 distinct scFv clones recognizing CXCL16 were identified and full-length Ab-11 were identified as the most potent CXCL6-neutralizing antibody, validating the library's efficiency for therapeutic antibody discovery.

## Results

### Design of the semisynthetic humanized scFv library with high diversity

**Figure 1:**
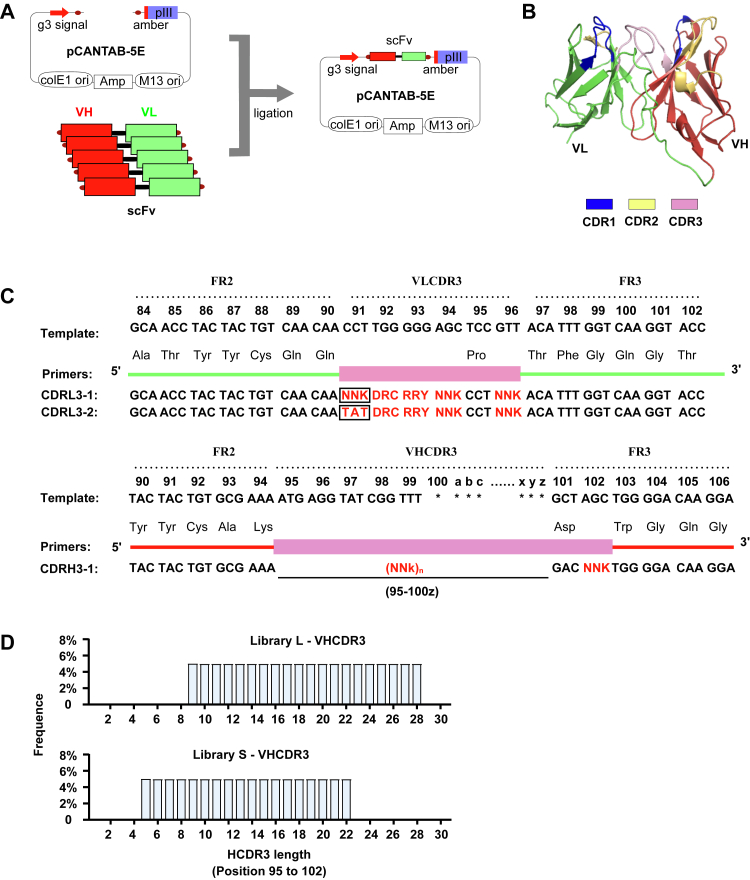
**Design of diversity in scFv repertoires.***A*, schematic of the recombinant phagemid vector (pCANTAB-5E) construction. *B*, structure of scFv . Heavy chain (*red*), light chain (*green*), and CDRs(*blue*, *yellow*, *light pink*) are indicated. *C*, design of scFv library CDR3 cassettes, amino acids are numbered by the Kabat rule, N = A/T/C/G, K = G/T, D = A/G/T, R = A/G, Y=C/T. *D*, length diversity design of the library CDR3. CDR, complementarity-determining region; scFV, single-chain variable fragment.

To obtain a highly reliable and diverse humanized scFv phage display library for specific antibody screening, we chose pCANTAB-5E phagemid vector to construct recombinant scFv (Fig. 1, *A* and *B*). This vector includes multiple components for phage display and exhibits high efficiency in transformation and display (36, 37). Prior research indicates that the VH3-Vκ1 antibody gene pairing is one of the most prevalently employed combinations within the human immune repertoire (38, 39, 40). Thus, we engineered the CDR3 region in accordance with the VH3-Vκ1 gene framework. Within the VL domain, amino acids at positions 91 to 96, which constitute the CDR3 loop, exhibit substantial diversity and play a role in determining the antigen recognition capacity of CDR3 (39, 41). Notably, position 91 in VL is relatively conserved and usually occupied by tyrosine (∼50%), and position 95 is highly conserved as proline (39). Therefore, to better mimic the amino acid composition of humanized antibodies, we strategically designed two primers to accommodate the high frequency of tyrosine at position 91 within the VL CDR3 region, while maintaining the conserved proline at position 95 without introducing additional mutations (Fig. 1*C*). In addition, to precisely control the limited diversity at positions 92 and 93, we incorporated oligonucleotides with DRC and RRY degenerate codons, respectively (Fig. 1*C*). For position 94 and 96, we employed the NNK degenerate codon to introduce all the amino acid types to ensure the rich diversity at these two positions (Fig. 1*C*). These degenerate codons enable mixed-base incorporation (N = A/T/C/G; K = G/T; D = A/G/T; R = A/G; Y = C/T), allowing tailored amino acid variability at each site. In the VH domain, a stabilizing salt bridge forms between the conserved aspartic acid at position 101 and arginine at position 94, which is critical for maintaining the VH CDR3 conformation. The intervening residues (95–100z) are crucial for antigen recognition and binding, providing abundant diversity and accommodating CDR3 regions of varying lengths (38, 39). Based on this structural understanding, we incorporated various numbers of NNK residues between the conserved arginine at position 94 and aspartic acid at position 101 in VH CDR3 region (Fig. 1*C*). In order to cover all length ranges, we constructed two sublibraries with different VH CDR3 lengths: the L (long) library (9–28 AA) and the S (short) library (5–22 AA). (Fig. 1*D*).

### Improving the mutagenesis efficiency of the Kunkel method

**Figure 2:**
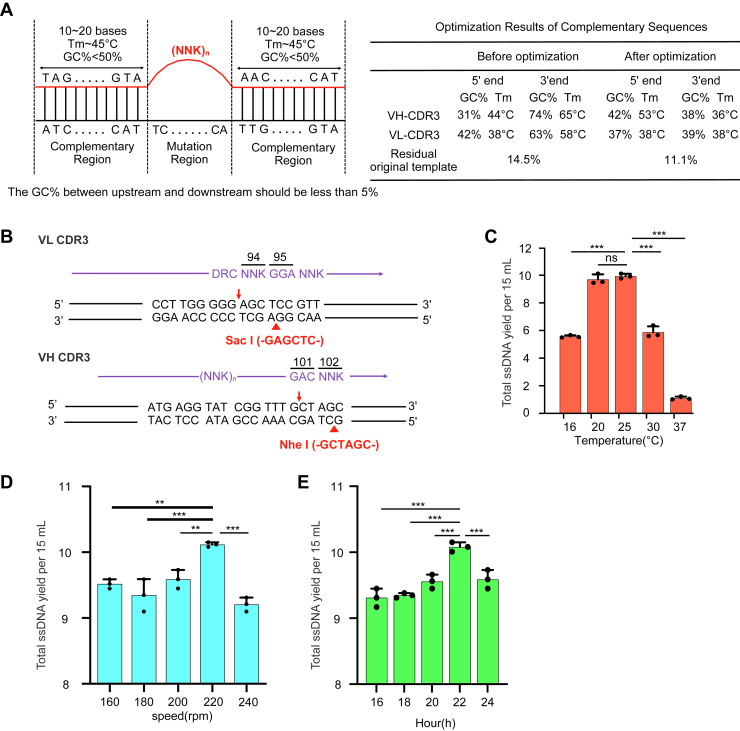
**Optimization of the Kunkel mutagenesis method.***A*, codon optimization of the primer upstream and downstream complementary regions (*left*) adjusted the GC content and Tm to minimize the original template residual (*right*). *B*, introduction of two unique restriction sites in VL and VH CDR3 region. *C*-*E*, the ssDNA production extracted from 15 ml CJ236 cell culture at different culture conditions including temperature (*C*), rotational speed (*D*), and culture time (*E*). Data are represented as means ± SD (n = 3), ∗∗*p* < 0.01, ∗∗∗*p* < 0.001; ns, not significant; One-way ANOVA followed by Tukey’s multiple comparisons test. CDR, complementarity-determining region; scFV, single-chain variable fragment; VH, variable domains of the heavy chain; VL, variable domains of the light chain.

In Kunkel’s method, the *Escherichia coli* CJ236 strain (dut^-^ ung^-^) serves as a critical host for site-directed mutagenesis and phage-display library construction. Inactivation of dUTPase (*dut*^*-*^) in CJ236 increases intracellular dUTP pools, leading to uracil substitution in the template strand, while uracil (U)-DNA glycosylase deficiency (*ung*^*-*^) prevents its excision. This allows selective degradation of the U-containing template in *ung*^*+*^ host cells (such as TG1 strain), enriching for mutant strands during library synthesis. However, this method still suffers from 20 to 30% residual template retention and suboptimal mutagenesis efficiency, complicating downstream screening and requiring further refinement (42). To address this problem, we first performed codon optimization for upstream and downstream complementary regions of the primers to ensure balanced GC content (≤5% difference), reduced GC content below 50%, and adjusted the melting temperature (Tm) closely to 45 °C (43). We also optimized the ratio between template primers and used gradient annealing instead of single annealing step (Table S1). These modifications increased the diversity of antibody library and reduced the original template content from 14.5% to 11.1% (Fig. 2*A*). Furthermore, to fully eliminate the original template, we introduced two unique restriction sites: SacI in VL CDR3 and NheI in VH CDR3 to enable subsequent efficient enzymatic excision of nonmutated templates (Fig. 2*B*). On the other hand, we improved the single-stranded DNA (ssDNA) production by optimizing CJ236 culture parameters. The results showed that incubating CJ236 cells at 20 °C or 25 °C with an agitation speed of 220 rpm and extending incubation to 22 h significantly improved ssDNA yield and quality (Fig. 2, *C*–*E*).

### Construction and diversity analysis of the humanized antibody library

**Figure 3:**
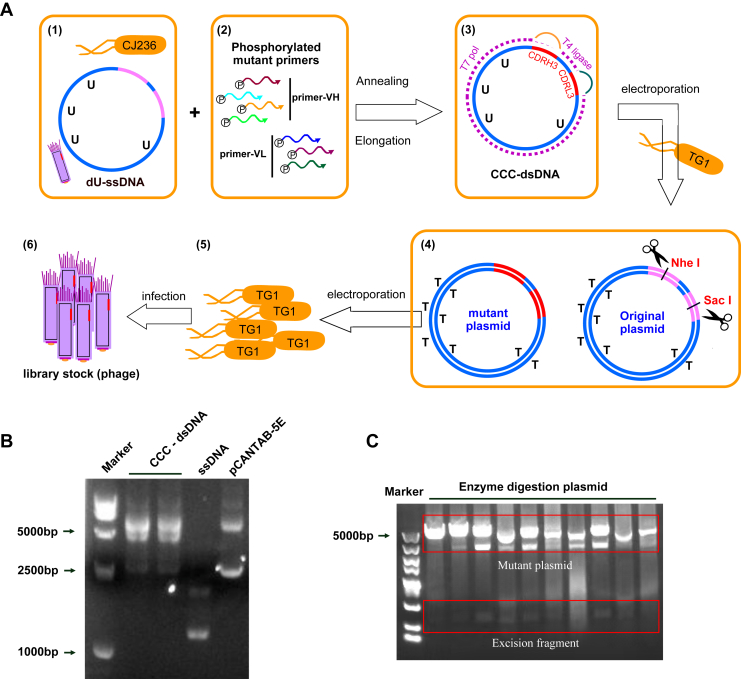
**Construction of a humanized scFv antibody library.***A*, stepwise schematic of the scFv phage display library construction. Step 1, prepared dU-ssDNA template using CJ236 cells; step 2, primer cassettes encoding VH and VL CDR3 with designed diversity were phosphorylated and annealed to the template to generate CCC-dsDNA; step 3, mutagenized dsDNA were electroporated into TG1 cells to break down the U-containing template; step 4, deplete the original template plasmid using the restriction enzymes; step 5 and 6, the mutant plasmid was re-electroporated into TG1 cells and packaged into phage particles to generate the final phage antibody library. *B*, the electrophoretic gel for the generated ssDNA and dsDNA in the construction process. *C*, the electrophoretic gel for restriction enzyme digested product, the original template was excised into small fragments (*bottom box* indicated), the mutant plasmid is uncut (*top box* indicated). CDR, complementarity-determining region; scFV, single-chain variable fragment; VH, variable domains of the heavy chain; VL, variable domains of the light chain.

Based on the aforementioned design and optimization strategies, we successfully constructed a highly diverse semisynthetic humanized phage display antibody library (Fig. 3*A*). In brief, pCANTAB-5E plasmid containing human scFv was transformed into CJ236 cells. Then the CJ236 cells were infected with helper phage to generate dU-ssDNA (uracil-containing single-stranded DNA) (Fig. 3*B*). Subsequently, *in vitro* phosphorylation was performed to synthesize heteroduplex covalently closed ds-DNA (CCC-dsDNA) incorporating U bases (Fig. 3*B*). The synthesized CCC-dsDNA was then electroporated into TG1 cells, enabling selective degradation of the U-containing template strand. The residual original template was further excised by the restriction enzyme digestion (Fig. 3*C*), and the mutant plasmids were secondly electroporated into TG1 cells, ultimately yielding the final phage display antibody library.

**Figure 4:**
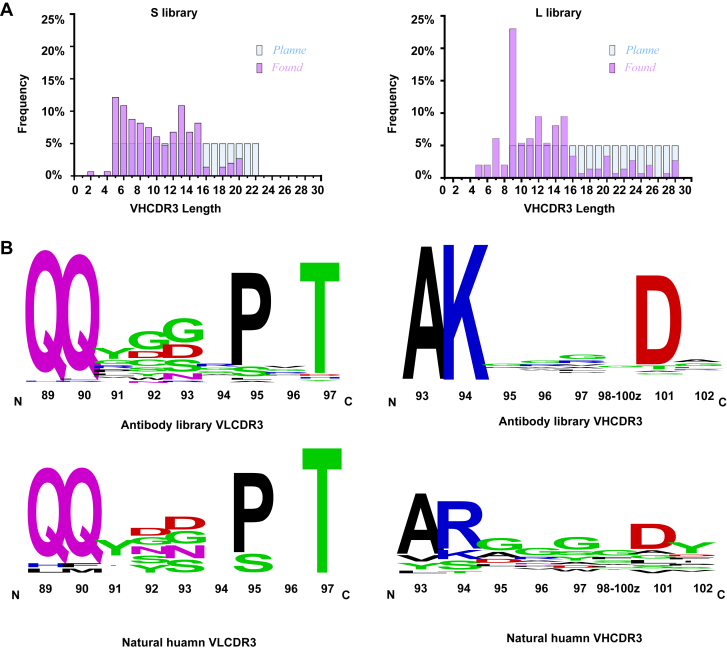
**Diversity analysis of the antibody library.***A*, distribution of VH CDR3 length variants in the S and L library. *B*, diversity distribution of amino acid sequences in constructed antibody library and natural human antibody CDR3 regions. CDR, complementarity-determining region; VH, variable domains of the heavy chain.

Through quantitative bacterial colony analysis, the size of the phage display antibody library was determined as 1.73 × 10^10^. The total colony numbers of the L and S sublibraries were 1.57 × 10^10^ and 6.93 × 10^9^, respectively. To assess the diversity of the library, 190 randomly selected colonies were subjected to sequencing the VH CDR3 and VL CDR3 regions. No original template was detected in the sequencing results, demonstrating the effectiveness of our restriction enzyme-based original template depletion strategy. The VH CDR3 length distribution analysis revealed distinct patterns in the two sublibraries. The S library exhibited a predominant range of 5 to 15 amino acids (all variants ≤ 20 AA) in the mutated region, whereas the L library showed a primary distribution of 9 to 16 amino acids, along with a small but substantial population of longer variants (17–28 AA) (Fig. 4*A*). These results demonstrated a broad length distribution of VH CDR3, although some deviations from the intended design were observed. This is likely due to the inherent stochastic nature of the mutagenesis process and the unpredictable incorporation of amino acids at specific positions. Comparative sequence analysis further revealed substantial homology in the light and heavy chain CDR3 regions between our constructed library and the natural human variants (Fig. 4*B* and Table S3). The intentional mutagenesis at selected nonconserved positions generated sequence variation that diverged from natural humanized antibody profiles while maintaining structural integrity. This targeted mutagenesis strategy was designed to maximize antibody diversity while preserving critical human immunoglobulin frameworks, thereby achieving an optimal balance between sequence variability and low immunogenicity.

### Screening of anti-CXCL16 antibodies from the constructed phage display library

**Figure 5:**
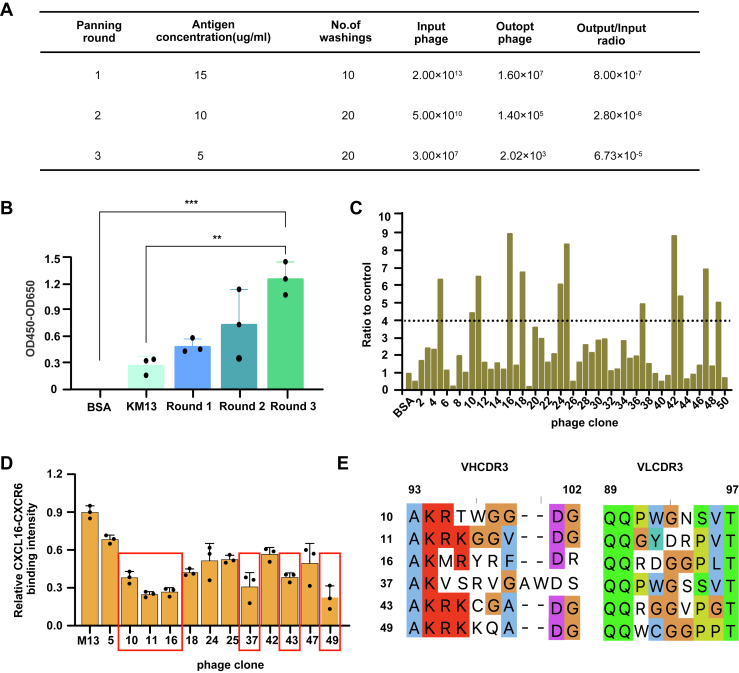
**Screening of anti-CXCL16 specific antibodies.***A*, panning efficiency for phage library enrichment in each round. *B*, polyclonal phage ELISA screening for CXCL16 binding. Data are represented as means ± SD (n = 3), ∗∗*p* < 0.01, ∗∗∗*p* < 0.001; One-way ANOVA followed by Tukey’s multiple comparisons test. *C*, monoclonal phage ELISA screening for CXCL16 binding after the third round of selection. *D*, flow cytometric analysis of the binding of CXCL16 protein to CXCR6-expressing 293T cells in the presence of the indicated phage scFv or M13 helper phage. Data are represented as means ± SD (n = 3). *E*, CDR3 sequence alignment of selected clones. CDR, complementarity-determining region; CXCL16, CXC motif chemokine ligand 16.

To evaluate the potential of our constructed phage display library, we chose chemokine CXCL16 as the target antigen for biopanning selection. CXCL16 is a multifunctional chemotactic cytokine that binds to CXCR6 on immune cells, playing critical roles in inflammation, tumor immune evasion, and tissue homeostasis (44, 45, 46). We conducted three rounds of panning against CXCL16 using our phage display library and acquired significantly enriched CXCL16-binders (Fig. 5, *A* and *B*). We randomly selected 50 monoclones for further ELISA-based binding analysis. Twelve candidates showed pronounced specificity, with *A*_450_ values exceeding negative control levels by ≥ 4-fold, indicating a high panning efficiency for our library (Fig. 5*C*).

To further validate the library’s potential for pharmaceutical applications, we generated a stable CXCR6-expressing cell line and performed flow cytometric analysis to evaluate antibody-mediated blockade of CXCL16-CXCR6 interaction. Six monoclonal scFvs exhibited potent inhibitory activity in soluble binding assays (Fig. 5*D*). Sequence analysis revealed that these monoclones shared similar CDR3 sequences except clone 37, which contained a nine AA insertion in the VH CDR3 region (Fig. 5*E*).

### Functional validation of anti-CXCL16 full-length antibodies

**Figure 6:**
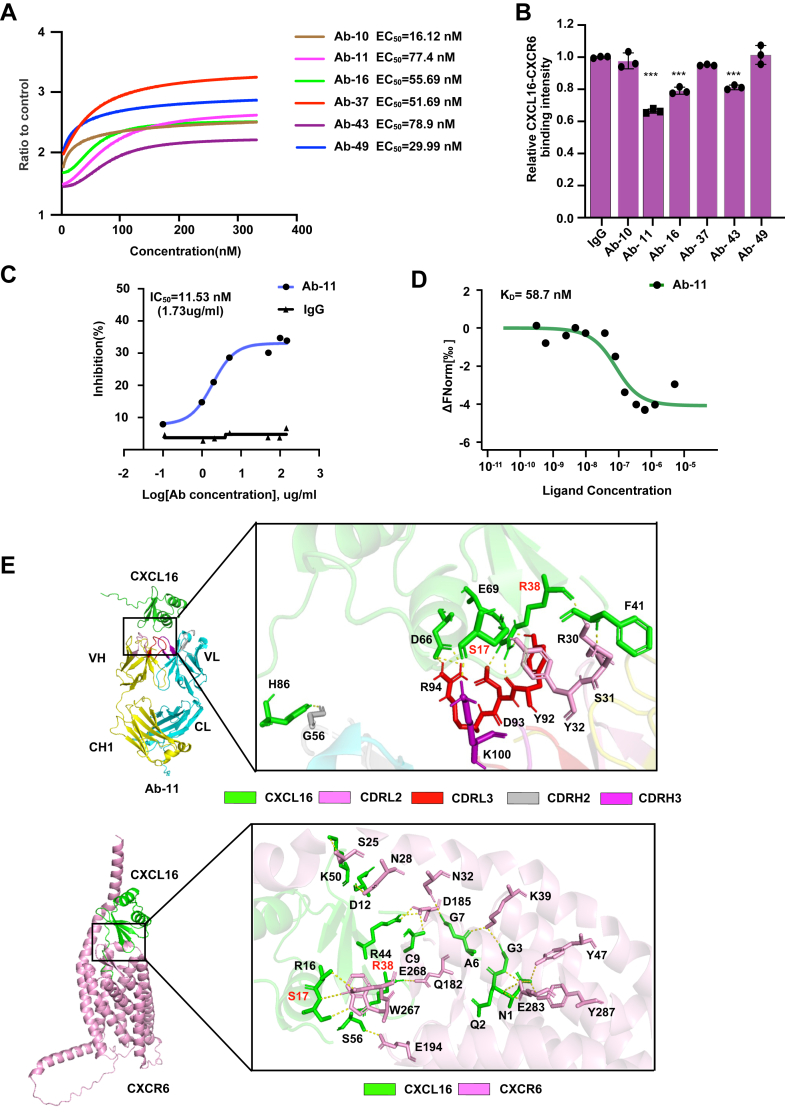
**Functional validation of anti-CXCL16 full-length IgG antibodies.***A*, ELISA analysis of the binding capacity for CXCL16 and purified antibodies with different concentrations. *B*, soluble ligand binding assay analyzed the neutralization activity of purified antibodies. Data are represented as means ± SD (n = 3). ∗∗*p* < 0.01, ∗∗∗*p* < 0.001; One-way ANOVA followed by Tukey’s multiple comparisons test. *C*, inhibitory effect of Ab-11 with different concentrations by soluble ligand binding analysis. Data are represented as means ± SD (n = 3). *D*, binding kinetics of Ab-11 by MST, data are represented as means ± SD (n = 3). *E*, the CXCL16–Ab-11 complex structure was predicted through ClusPro 2.0, and key interaction residues were identified. CXCL16, CXC motif chemokine ligand 16; IgG, immunoglobulin G; MST, microscale thermophoresis.

Next, the six CXCL16-blocking antibody candidates were converted into full-length immunoglobulins G (IgGs) and expressed in 293T cells. ELISA analysis showed that all these purified full-length antibodies displayed specific binding affinity to CXCL16 (EC_50_ = 15–80 nM) (Fig. 6*A*). Soluble ligand binding analysis further demonstrated that only Ab-11, Ab-16, and Ab-43 retained CXCL16-CXCR6 blocking activity, while the other antibodies showed no significant inhibitory effect (Fig. 6*B*). This might be due to the multivalent avidity effects and greater steric hindrance for phage displayed scFvs (47, 48). The conformation of the CDR may also change when converted into full-length antibodies that disrupt critical epitope interactions required for CXCL16 blockade (49, 50, 51). Among three blocking antibodies, Ab-11 exhibited the most potent inhibitory activity, with a semiinhibitory concentration (IC_50_) of 11.53 nM (Fig. 6*C*) and a dissociation constant (K_D_) of 58.8 nM (Fig. 6*D*), indicating superior binding affinity and functional blockade of CXCL16-CXCR6 interaction.

Through protein-protein docking analysis, we found that Ab-11 sterically blocks the CXCL16-CXCR6 binding interface. S17 and R38 of CXCL16 are critical binding sites both for CXCL16 - Ab-11 and CXCL16-CXCR6 interactions (Fig. 6, *E* and *F*). These results elucidate the potential molecular mechanism underlying the neutralization of Ab-11 for CXCL16.

## Discussion

Antibody therapeutics have transformed modern medicine, with over 200 approved clinical treatments and approximately 1400 candidates currently in development (52). However, the growing demand for antibody-based treatments has underscored critical challenges, including the need for improved production efficiency, reduced immunogenicity, and enhanced stability (10). The synthetic antibody generation strategy provides distinct advantages, positioning it as a promising solution to these challenges. In this study, we present a convenient and feasible human antibody library construction platform by improving the traditional Kunkel’s method and construct a highly functional library with the size approximately achieving 1.7 × 10^10^. The capacity of this library surpasses that of most commercial antibody libraries and those previously reported in the literature. Furthermore, the library exhibits broad sequence and length diversity in both the VH CDR3 and VL CDR3 regions, closely recapitulating the natural human antibody repertoires. This design achieves exceptional diversity while preserving the structural stability and developability characteristic of naturally derived antibodies, enabling more efficient screening of high-affinity antibodies and identification of rare epitopes.

A key aspect of our methodology was the optimization of the Kunkel mutagenesis protocol, which has been constrained by low efficiency and residual original template contamination. Through refining the culture parameters, primer design and annealing conditions, we significantly improved the mutagenesis efficiency and minimized the original template content. These advancements not only enhance the diversity and quality of our library but also provide a reproducible framework for synthetic library construction. Notably, the strategic introduction of restriction sites within the CDR3 regions enabled complete original template removal without compromising the library diversity, representing a key improvement over conventional approaches.

CXCL16 plays a pivotal role in inflammation, fibrosis, and tumorigenesis by mediating immune cells recruitment (35, 45, 53, 54). Therapeutic intervention targeting CXCL16 represents a promising strategy for these diseases. Preclinical studies have demonstrated that CXCL16-targeting biologics (*e.g.*, XOMA-089 and various bispecific antibodies) exhibit therapeutic potential in multiple diseases, such as rheumatoid arthritis, atherosclerosis, and cancer immunotherapy (32, 44, 55). However, the clinical translation still faces challenges from pathway redundancy and safety concerns. Therefore, developing improved CXCL16-targeting antibodies remains an urgent priority. In our study, we successfully screened out 12 significant binders to CXCL16 from 50 randomly selected clones. Six of them were converted into full-length antibodies and exhibit specific and high affinity to CXCL16, indicating an excellent panning efficiency for our library. Furthermore, we identified Ab-11 as a highly potent CXCL6-neutralizing antibody (IC_50_ 11.53 nM, K_D_ 58.8 nM), which can sterically block the CXCL16-CXCR6 binding interface, providing a molecular basis for rational antibody optimization.

In summary, our study establishes a robust and high-efficiency platform for constructing a highly diverse and functionally validated humanized scFv library. By integrating optimized Kunkel mutagenesis design and technological process, we developed a scalable method that can be broadly applied to library construction and antibody discovery, offering significant potential for advancing therapeutic development.

## Experimental procedures

### Construction of recombinant phagemid vector

The pCANTAB-5E vector (Kelei Biotechnology) and humanized scFv gene (from laboratory constructed plasmid pIT2-scFv) were digested using SfiI and NotI restriction enzymes (NEB). Following digestion, the fragments were analyzed by 1% agarose gel electrophoresis, and appropriately sized bands were purified using the Universal DNA Purification Kit (TIANGEN). The digested vector and insert fragments were then ligated using T4 DNA ligase (NEB), and the resulting constructs were transformed into *E. coli* DH5α strain. Single clones were selected the next day and verified by DNA sequencing.

### Primer synthesis and pool preparation

All primers were synthesized by Qingke. Detailed synthesis information can be found in Table S2. The mass of the phosphorylated primer was 0.6 μg (as shown in Table S1). Based on the known relationship: n = c/v = N/N_A_ and N = cN_A_/v, we diluted all primers to a concentration of 100 μM. By adding the same volume of each primer, we prepared a pool containing equimolar primer molecules.

### Helper phage amplification

A single TG1 colony was inoculated into 20 ml of 2YT medium and grown overnight at 37 °C with shaking (220 rpm). A 1% inoculum was transferred to 20 ml of 2YTG medium (1% glucose) and cultured until *A*_600_ reached 0.7. Helper phages were added, and the culture was incubated for 1h at 37 °C. The infected cells were then expanded into 200 ml of 2YTK medium (0.5% kanamycin) and incubated overnight (37 °C, 220 rpm). The culture was centrifuged (12,000*g*, 15 min, 4 °C), and the supernatant was collected and recentrifuged (12,000*g*, 10 min, 4 °C) to remove residual cell debris. Phages were precipitated by adding PEG/NaCl (1:5 v/v) and incubated on ice for ≥ 4 h, followed by centrifugation (12,000*g*, 15 min, 4 °C). The pellet was resuspended in 20 ml PBS, reprecipitated with PEG/NaCl (1:5 v/v) for ≥ 1 h, and centrifuged again (12,000*g*, 20 min, 4 °C). The final pellet was resuspended in PBS for downstream applications.

### Extraction and phosphorylation of single-stranded DNA

Recombinant phagemids were transformed into *E. coli* CJ236 (Takara), and individual colonies were grown overnight in 2YT medium (0.1% ampicillin and 0.1% chloramphenicol) at 37 °C with shaking (220 rpm). A 1% inoculum was transferred to fresh 2YT medium and cultured until *A*_600_ reached 0.6, followed by infection with helper phage (multiplicity of infection ∼10–20) for 1 h at 37 °C (200 rpm). Cells were centrifuged (5000*g*, 20 min), resuspended in 100 ml 2YT medium (0.1% ampicillin, 0.5% kanamycin, and 0.1% chloramphenicol), and incubated at 25 °C for 20 to 24 h. The culture supernatant was collected by centrifugation (12,000*g*, 20 min), and phage particles were precipitated with PEG/NaCl (ice bath, 30 min), centrifuged (12,000*g*, 30 min, 4 °C), and resuspended in 2 ml PBS. After debris removal (12,000*g*, 5 min), purified phages were stored at 4 °C. The ssDNA was extracted using the E.Z.N.A. M13 DNA Isolation Kit (Omega) per the manufacturer’s protocol. The experimental setup and protocol for *in vitro* phosphorylation are detailed in Table S1 (56).

### Preparation and electrotransformation of electroreceptive cells

TG1 cells were inoculated into 10 ml of 2YT medium and grown overnight at 37 °C with shaking at 220 rpm. The culture was then diluted 1:100 into 1 L of fresh 2YT medium and incubated until the *A*_600_ reached 0.7. The bacterial suspension was divided equally into two prechilled 500 ml centrifuge tubes (Thermo Fisher Scientific) and incubated on ice for 30 min. Cells were pelleted by centrifugation at 5000*g* for 5 min at 4 °C, resuspended in 50 ml of ice-cold glycerol/Hepes buffer, and recentrifuged at 5000*g* for 15 min at 2 °C. The final pellet was gently resuspended in 1 ml of ice-cold glycerol/Hepes to obtain electrocompetent cells.

The BTX electroporation cuvette was removed and rinsed 5 to 10 times with deionized water to remove residual alcohol. After inversion for air-drying, it was placed on ice. The super optimal broth with catabolic repressor (SOC) medium was preheated to 37 °C and competent cells/plasmids were cooled on ice. Plasmids were mixed with cells, transferred to an electroporation cuvette, dried by brief centrifugation (to remove bubbles), and electroporated at optimized voltage. Post electroporation, 1 ml preheated SOC medium was added, followed by transfer to 12 ml SOC medium, and 1 h incubation (37 °C). Transformation efficiency was measured after dilution.

### Analysis of sequence diversity

After electroporation, the transformed bacteria were serially diluted, and the library size was quantified through colony counting (n = 3 technical replicates per dilution). Randomly selected clones underwent scFv fragment amplification, followed by sequence analysis. Sequence and length diversity of the antibody library were statistically evaluated with sequence diversity profiles visualized using the online Weblogo platform (http://weblogo.berkeley.edu).

### Screening of specific antibodies

The immunotube was coated overnight with 4 ml of CXCL16, then washed three times with PBS the next day. It was subsequently filled to the brim with 2% milk-PBS, incubated at room temperature, and washed three times with PBS. For phage panning, 10^12^-10^13^ phages in 4 ml of 2% milk-PBS were added to the tubes and incubated for 1 h with rotation, followed by 1 h of static incubation at room temperature. Unbound phages were removed by washing 10 to 20 times with PBS containing 0.1% Tween-20. Bound phages were eluted by adding 500 μl of trypsin-PBS solution and incubating the mixture with rotation at room temperature for 10 min. The eluted phage (250 μl) was mixed with 1.75 ml of TG1 cells (*A*_600_ = 0.4) and incubated without agitation in a 37 °C water bath for 30 min. After infection, the bacterial suspension was diluted for phage titer determination, while the remaining culture was centrifuged (11,600*g*, 5 min) and plated on TYE agar containing 100 μg/ml ampicillin and 1% glucose. Following overnight incubation at 37 °C, colonies were resuspended in 2 ml of 2 × TY medium with 15% glycerol using a glass spreader. For phage amplification, 50 μl of this suspension was inoculated into 50 ml of 2 × TY medium (100 μg/ml ampicillin, 1% glucose) to generate phage for subsequent screening rounds.

### Cell transfection

Subsequently, 293T cells (American Type Culture Collection) at 60 to 70% density were treated with 25 μM chloroquine 1 h before transfection. The transfection mixture was prepared by mixing 10 to 15 μg pCDH-CXCR6 plasmid (in 1095 μl total volume) with 155 μl 2 M CaCl_2_, followed by dropwise addition of 1250 μl 2×HBS with gentle vortexing. The mixture was immediately added to cells within 1 to 2 min, ensuring even distribution. The medium was replaced with fresh medium 7 to 12 h post transfection. CXCR6 expression was analyzed 48 h after transfection.

### Purification of full-length IgG antibodies

The supernatant from transfected 293T cells was centrifuged at 1200 rpm for 3 min to remove debris, then diluted with an equal volume of binding buffer under continuous stirring. The mixture was adjusted to pH 8.0 at 4 °C, centrifuged at 15,000 rpm for 30 min, and the clarified supernatant was loaded onto a Protein A column. The column was washed with 15 column volumes of binding buffer, and the target protein was eluted with 10 column volumes of elution buffer into collection tubes pretreated with neutralization buffer (1:10 ratio to elution volume). The eluent was concentrated *via* centrifugal filtration, buffer-exchanged into PBS, and stored at −80 °C.

### Soluble ligand binding

Phages were diluted to a concentration of 10^12^/ml, and 40 μl was mixed with CXCL16 at a final concentration of 50 ng/μl in a total volume of 50 μl, adjusted with ddH_2_O if needed. After thorough mixing, the mixture was incubated at 4 °C for 1 h, followed by the addition of anti-His antibody (BioLegend), and a second incubation for 1 h at 4 °C. Concurrently, cells were detached using trypsin, washed 2 to 3 times with PBS, and fixed with 2% paraformaldehyde at room temperature for 15 min. After fixation, cells were washed again with PBS, and the 50 μl mixture was added to the cells and incubated on ice for 30 min. Cells were then washed once with PBS, resuspended in 1 ml PBS, filtered through a 70-μm mesh, and analyzed by flow cytometry.

### ELISA

For the phage ELISA, 96-well plates (Greiner Bio-One) were coated with CXCL16 at a concentration of 5 μg/ml per well. Subsequently, 100 μl of phage, diluted in 2% milk in PBS, was added to each well and incubated for 1 h. The ELISA was developed using HRP-conjugated anti-M13 (Sino Biological) and tetramethylbenzidine (Sino Biological) as the substrate.

### Molecular modeling docking

Structural simulations were performed using AlphaFold 3 (https://alphafoldserver.com/fold/7718756da53e9fbb) and IgG Modeling (https://wemol.wecomput.com/ui/#/). Antibody-antigen docking was conducted using ClusPro 2.0 (https://cluspro.bu.edu/results.php). The resulting complexes were analyzed using PyMOL (v2.6.0).

### Statistical analysis

Statistical significance was determined by one-way ANOVA (GraphPad Prism 10.0) after verifying homogeneity of variances with the Brown-Forsythe test. The data were obtained from at least three independent replicates and are presented as means ± standard deviation (SD). *p* values are denoted as follows: ns (not significant), ∗∗*p* < 0.01, ∗∗∗*p* < 0.001.

## Data availability

The data supporting the findings of this study are available within the article and in its supporting information. This article contains supporting information.

## Supporting information

This article contains supporting information (39, 43).

## Conflict of interest

The authors declare that they have no conflicts of interest with the contents of this article.
