# Supplementary material for: A diverse semisynthetic humanized scFv phage display library for anti-CXCL16 antibodies
Source: J Biol Chem. 2025 Sep 8;301(10):110692. doi: 10.1016/j.jbc.2025.110692 (PMC12528905; doi:10.1016/j.jbc.2025.110692)
Supplement: Supporting Tables [file mmc1.pdf]

A diverse semi-synthetic humanized scFv phage display library for anti-CXCL16 antibodies

ZhenSheng Li, Qi Chen, Shihui Wang, JianFeng Chen,* ShiYang Chen*

*Correspondence:chenshiyang2014@sibcb.ac.cn, jfchen@sibcb.ac.cn

This file includes the supplymently information：

Table S1 to S3

Table S1. The experimental setup and protocol for in vitro

Reaction 1. Oligonucleotide phosphorylation with T4 polynucleotide kinase		
Component	Amount	Finall
mutagenic oligonucleotides		0.6
10 xTM buffer	2ul	1x
10mM ATP	2ul	1mM
100mM DTT	1ul	5mM
T4 polynucleotides (10U/ul)	2ul	20U
ddH_2O	up to 20 ul	
	Reaction setting : 37°C for 1h	

Reaction 2. Annealing of the oligonucleotides to the template		
Component	Amount	Finall
dU-ss DNA template	20ug	20ug
10 xTM buffer	25ul	1x
phosphorylated CDRH3 oligonucleotides	20ul	0.6ug
phosphorylated CDRL3 oligonucleotides	20ul	0.6ug
ddH_2O	up to 250 ul	
	Reaction setting : 90°C for 3min;<br>65°C for 5min;<br>60°C for 5min;<br>55°C for 5min;<br>50°C for 5min;<br>20°C for 5min;	

Reaction 3. Enzymatic synthesis of CCC-dsDNA		
Component	Amount	Finall
annealed oligonucleotides/template mixtures	250ul	
10mM ATP	10ul	
dNTP mix(25mM of each nucleotide)	10ul	
100mM DTT	15ul	5mM
T4 DNA ligase	1ul	30 Weiss units
T7 DNA polymerase	3ul	30U
	Reaction setting : 20°C for overnight	

Table S2. Synthetic primer

primer	sequences				Base
VL-1	TACCTTGACCAAATGT	MNNAGGMNNRYYGYHMNN		TTGTTGACAGTAGTAGGT	52
VL-2	TACCTTGACCAAATGT	MNNAGGMNNRYYGYHATA		TTGTTGACAGTAGTAGGT	52
VH	TATTTGTTCCTTGTCCCCA	MNNGTC $(MNN)_{1\text{-}24}$	MNNMNN	TTTCGCACAGTAGTAT	50

Table S3. Comparative sequence analysis of CDR3 regions

VL

Kabat position	89		90		91		92		93		94		95		96		97	
**Codon**	CAG		CAG		TAT / NNK		DRC		RRY		NNK		CCT		NNK		ACG	
**Planned /Found**	P	F	P	F	P	F	P	F	P	F	P	F	P	F	P	F	P	F
A					3.10	6.14		1.75			6.25	9.65		5.26	6.25	6.14		
C					1.55	5.26	16.70	17.54		0.88	3.10	5.26			3.10	3.51		
D				0.88	1.55		16.70	14.04	25.00	17.54	3.10	1.75			3.10	0.88		2.65
E		0.88			1.55	0.88		0.88		0.88	3.10	0.88			3.10	2.63		
F					1.55						3.10	2.63			3.10	2.63		0.88
G					3.10	11.40	16.70	32.46	25.00	44.74	6.25	13.16			6.25	10.53		
H		1.75			1.55	0.88		0.88		0.88	3.10	0.88			3.10	1.75		0.88
I					1.55	0.88					3.10				3.10	1.75		
K					1.55						3.10	1.75			3.10			
L				2.63	4.70	7.89			9.40	10.53		3.51	9.40	8.77				
M							1.55				3.10	0.88			3.10			
N				0.88	1.55		16.70	4.39	25.00	10.53	3.10	0.88			3.10	0.88		
P					3.10	18.42					6.30	5.26	100	72.81	6.30	7.02		0.88
Q	100	96.49	100	93.86	1.55						3.10				3.10	2.63		
R		0.88		0.88	4.70	12.28		0.88		1.75	9.40	14.04		0.88	9.40	9.65		0.88
S					1.55		16.70	8.77	25.00	20.18	3.10	18.42		16.67	3.10	7.02		
T					3.10	0.88				0.88	6.30	3.51		0.88	6.30	0.88	100	92.92
V					3.10	5.26					6.30	6.14			6.30	24.56		
W					1.55	3.51		12.28		1.75	3.10	2.63			3.10	1.75		
Y				0.88	51.60	25.44	16.70	5.26			3.10	0.88			3.10	3.51		0.88
stop codon					1.56	0.88		0.88			3.13	0.88			3.13	3.50		

VH

Kabat position	93		94		95		96		97		98-100z		101		102	
**Codon**	GCG		AAA		NNK		NNK		NNK		NNK		GAC		NNK	
**Planned /Found**	P	F	P	F	P	F	P	F	P	F	P	F	P	F	P	F
A	100	100			6.25	8.33	6.25	8.33	6.25	9.17	6.25	9.72		3.33	6.25	13.33
C					3.10	5.83	3.10	4.17	3.10	5.00	3.10	4.58			3.10	1.67
D					3.10	2.50	3.10	3.33	3.10	2.50	3.10	2.01	100	83.33	3.10	1.67
E					3.10	1.67	3.10		3.10	2.50	3.10	1.23		0.83	3.10	0.83
F					3.10	2.50	3.10	4.17	3.10	3.33	3.10	5.47			3.10	5.83
G					6.25	16.67	6.25	18.33	6.25	15.00	6.25	15.75		4.17	6.25	13.33
H					3.10	1.67	3.10		3.10	1.67	3.10	1.01			3.10	5.00
I					3.10	2.50	3.10	2.50	3.10	2.83	3.10	1.23			3.10	2.50
K			100	100	3.10		3.10	1.67	3.10		3.10	0.11			3.10	0.83
L					9.40	5.00	9.40	7.50	9.40	9.17	9.40	10.50			9.40	9.17
M					3.10	7.50	3.10	0.83	3.10		3.10	0.89		0.83	3.10	0.83
N					3.10		3.10	0.83	3.10		3.10	0.45			3.10	0.83
P					6.30	5.00	6.30	5.00	6.30	6.67	6.30	7.15			6.30	5.00
Q					3.10		3.10	1.67	3.10		3.10	0.45			3.10	1.67
R					9.40	12.50	9.40	13.33	9.40	14.17	9.40	11.28		0.83	9.40	10.00
S					3.10	6.67	3.10	3.33	3.10	5.83	3.10	7.60		0.83	3.10	7.50
T					6.30	3.33	6.30	3.33	6.30	3.33	6.30	1.68		5.00	6.30	7.50
V					6.30	9.17	6.30	8.33	6.30	9.17	6.30	11.06			6.30	7.50
W					3.10	5.00	3.10	9.17	3.10	2.50	3.10	4.25		0.83	3.10	5.00
Y					3.10	1.67	3.10	2.50	3.10	5.83	3.10	1.56			3.10	0.83
stop codon					3.13	2.50	3.13	1.67	3.13	3.33	3.13	1.79			3.13	2.50

Table S3. Comparative sequence analysis of CDR3 regions. The CDR3 regions are numbered according to Kabat rule. The table summarizes the frequency of each amino acid at every position within VL and VH CDR3 regions.
